# A Study on the Performance of Vacuum Membrane Distillation in Treating Acidic, Simulated, Low-Level Radioactive Liquid Waste

**DOI:** 10.3390/membranes15070213

**Published:** 2025-07-18

**Authors:** Sifan Chen, Yan Xu, Yuyong Wu, Yizhou Lu, Zhan Weng, Yaoguang Tao, Jianghai Liu, Baihua Jiang

**Affiliations:** China Nuclear Power Engineering Co., Ltd., China National Nuclear Corporation, Beijing 100048, China; chensfb2025@126.com (S.C.);

**Keywords:** vacuum membrane distillation, low-level radioactive liquid waste, nitric acid

## Abstract

This study systematically explored the performance of a vacuum membrane distillation (VMD) system equipped with polytetrafluoroethylene (PTFE) hollow fiber membranes for treating simulated, acidic, low-level radioactive liquid waste. By focusing on key operational parameters, including feed temperature, vacuum pressure, and flow velocity, an orthogonal experiment was designed to obtain the optimal parameters. Considering the potential application scenarios, the following two factors were also studied: the initial nuclide concentrations (0.5, 5, and 50 mg·L^−1^) and tributyl phosphate (TBP) concentrations (0, 20, and 100 mg·L^−1^) in the feed solution. The results indicated that the optimal operational parameters for VMD were as follows: a feed temperature of 70 °C, a vacuum pressure of 90 kPa, and a flow rate of 500 L·h^−1^. Under these parameters, the VMD system demonstrated a maximum permeate flux of 0.9 L·m^−2^·h^−1^, achieving a nuclide rejection rate exceeding 99.9%, as well as a nitric acid rejection rate of 99.4%. A significant negative correlation was observed between permeate flux and nuclide concentrations at levels above 50 mg·L^−1^. The presence of TBP in the feed solution produced membrane fouling, leading to flux decline and a reduced separation efficiency, with severity increasing with TBP concentration. The VMD process simultaneously achieved nuclide rejection and nitric acid concentration in acidic radioactive wastewater, demonstrating strong potential for nuclear wastewater treatment.

## 1. Introduction

The effective treatment and safe discharge of low-level radioactive liquid waste (LLLW) represent critical components of radioactive waste management in nuclear facilities, directly influencing both operational safety and economy efficiency. The discharge of radionuclides in liquid effluents from nuclear fuel cycle facilities must meet the limits specified in GB 13695-1992 [1]. Other chemical indices of the effluents are a pH of 6–7 and a conductivity ≤20 μs·cm^−1^, which are derived from the environmental impact assessment results of the site where the nuclear fuel cycle facilities are located. The nuclear fuel cycle facilities adopt the PUREX process. During the actual operation of the plant, the LLLW from acidic processes still contains a high concentration (>0.1 mol·L^−1^) of nitric acid and low-level radionuclides. It is necessary to further remove these radionuclides from the LLLW to meet the discharge requirements, while simultaneously removing nitric acid. The concentrate generated during the purification process is subject to solidification treatment. If alkali is first added for neutralization to reduce the concentration of nitric acid, followed by reverse osmosis (RO) for radionuclide removal, a large amount of concentrate with a high salt content will be produced. After solidification, it will form a large amount of radioactive solid waste, which does not meet the requirement of waste minimization. If ion exchange resin or reverse osmosis is first used to remove radionuclides, the generated purified liquid is still acidic. After neutralization, the purified liquid has a high salt content, which cannot meet the conductivity requirements. Moreover, the exchange efficiency of the commercial resin is low in a strongly acidic environment, and frequent replacement will generate a large amount of radioactive waste resin [2]. The recommended operating condition for commercial reverse osmosis membranes is that the feed liquid is neutral, as the performance of reverse osmosis decreases in highly acidic environments [3,4]. This puts forward higher requirements for the treatment technology of LLLW from the acidic process, whereby the nuclides and nitric acid in the waste liquid should be concentrated simultaneously to form a small-volume concentrate with a low salt content. Then, the generated purified liquid meets the requirements without additional alkali neutralization and can be discharged into the environment.

Membrane distillation (MD) is an advanced separation process utilizing vapor pressure differences in volatile components across hydrophobic membranes; this process has emerged as a promising technology for LLLW treatment due to its high concentration efficiency, excellent separation performance, and mild operational conditions [5,6]. Extensive studies have been conducted to explore its applications in radioactive liquid management.

JIA et al. [7] constructed a pilot-scale VMD system with a treatment capacity of 0.8 m^3^·d^−1^ to treat simulated radioactive liquid containing cesium (Cs). The results indicated a stable decontamination factor (DF) of up to 110.3 for Cs, demonstrating consistent performance across various operational parameters. Similarly, LI et al. [8] evaluated a self-developed non-contact membrane distillation device for radioactive liquid treatment, achieving a strontium (Sr) DF of 4.6 × 10^6^ in simulated solutions. A further study from the same authors reported a total alpha/beta radioactivity reduction from 2 × 10^4^ Bq·L^−1^ to below 1 Bq·L^−1^ in actual radioactive feed solutions. JIA’s work also confirmed VMD rejection rates exceeding 99.6% for both Sr and cobalt (Co) [9,10]. Wen et al. investigated direct contact membrane distillation (DCMD) for high-salinity radioactive wastewater treatment, observing that the nuclide DF exceeded 10^5^ even at elevated salt concentrations [11]. Overall, these studies confirm the effective purification capabilities of MD technologies for LLLW.

Notably, recent investigations have expanded MD applications to acid recovery systems. The technology has demonstrated that MD could condense the non-volatile acids (e.g., H_2_SO_4_ and H_3_PO_4_) to a 40% concentration, while maintaining high rejection rates for volatile acids (e.g., HCl and HNO_3_) within specific concentration ranges [12,13,14,15]. These research results indicate that MD not only exhibits a high DF for nuclides but also achieves a certain concentration efficiency for nitric acid, demonstrating potential in treating radioactive wastewater containing nitric acid.

This study evaluated the performance of vacuum membrane distillation (VMD) for treating simulated acidic radioactive wastewater using a laboratory-scale setup. Orthogonal experiments were conducted to determine the optimal operation parameters, confirming the feasibility of VMD for acidic radioactive wastewater treatment. The effect of varying nuclide concentrations and tributyl phosphate (TBP)—a typical radioactive waste contaminant—on VMD process efficiency was also analyzed.

## 2. Materials and Methods

### 2.1. Experimental Device and Materials

**Figure 1:**
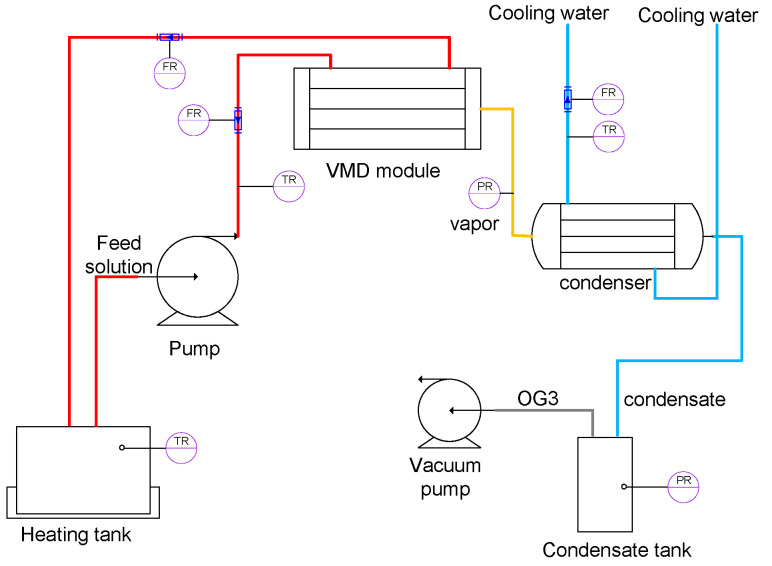
Schematic diagram of the VMD device.

A schematic diagram of the VMD device utilized in the experiment is presented in Figure 1. This device primarily comprises the following components:

1. A hot-side distillation source system, including a water bath heating tank, a high-temperature corrosion-resistant variable frequency pump (0.6T/H-12M, LiYu Energy Saving Equipment Co., Ltd., Foshan, China), a high-temperature corrosion-resistant flow meter (LZM-6T, Jintai Instrument Co., Ltd., Yuyao, China), a corrosion-resistant temperature probe and a multi-channel temperature recorder(TCP-500XL-8, Huipu Instrument Co., Ltd., Zhongshan, China), pipes, valves, and insulation.
2. A cold-side condensation system, including a condenser tube, a chilled water circulation loop with thermal insulation, and a condensate collection vessel.
3. A vacuum generation system, comprising a water-circulating vacuum pump (SHB-III, Zhengzhou Great wall Scientific Industrial & Trade Co., Ltd., Zhengzhou, China).
4. A metering system equipped with a digital transmission electronic scale (RS232 signal interface, OHAUS, Parsippany, NJ, USA).

The VMD module utilized in this study is a polytetrafluoroethylene (PTFE) hollow fiber membrane bundle with the specifications of φ50×370-BS. The VMD module was provided by Zhejiang Dongda Environment Company (Zhuji, China), and the internal PTFE membrane filaments were self-produced by the company. The key membrane characteristics include the following:

1. Effective membrane surface area: 0.3 m^2^;
2. Membrane dimensions: 1.2 mm inner diameter and 2.3 mm outer diameter;
3. Pore size distribution: 0.1 μm nominal pore diameter;
4. Pressure: 0.1 MPa bubble point pressure.

**Figure 2:**
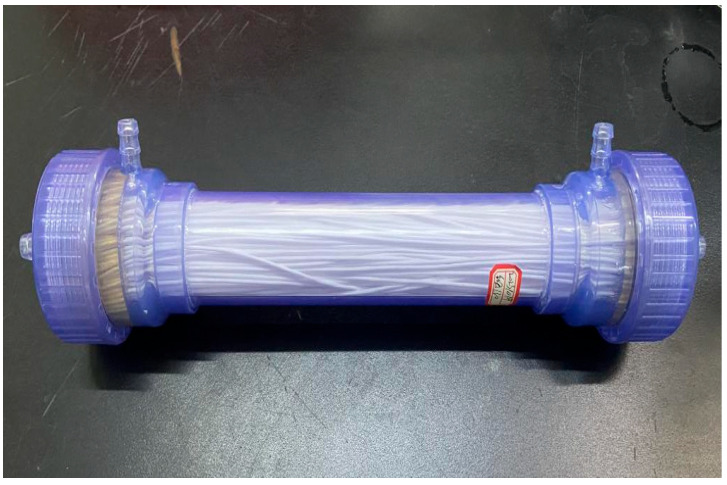
VMD module.

The VMD module is presented in Figure 2.

### 2.2. Experimental and Analytical Methods

Prior to the system startup, the feed solution was preheated to the target temperature using a water bath heating unit. A water-circulating vacuum pump was then activated to establish sub-atmospheric pressure in the permeate chamber. The heated feed solution was subsequently pumped through the VMD module at pre-set flow rates using a variable-frequency pump, forming a closed-loop hot-side circulation system. The retentate stream, after traversing the membrane module, was recirculated back to the feed vessel. Concurrently, vapor from the feed solution permeated through the hollow fiber membrane’s microporous structure, condensed in the cooled condenser coil, and was collected as a distillate in the product water tank.

In the experiment, the pH of the obtained samples was determined using a pH meter (LeiCi, Shanghai, China). The concentrations of nuclides in the feed solution and the distillate were quantified using Inductively Coupled Plasma–Mass Spectrometry (ICP–MS, Thermo Fisher Scientific, Waltham, MA, USA). To assess the alteration in the hydrophilicity of the membrane surface after wetting and fouling, the contact angle of the membrane surface was measured with a contact angle meter (Model SL200KS, KINO, New York, NY, USA). Moreover, a scanning electron microscope (SEM, Model SU8000, Hitachi, Tokyo, Japan) was employed to observe the surface and cross-sectional morphologies of the membrane after wetting and fouling.

The membrane flux (*J*) is a critical parameter in evaluating membrane performance; it can be calculated using the following formula:

(1) $$J=\frac{\Delta m}{\Delta t\cdot A\cdot \rho }$$

*J*: Membrane flux, typically expressed in L·m^−2^·h^−1^;

*Δm*: Permeate volume (kg);

*A*: Effective membrane area (m^2^);

*Δt*: Filtration time interval (h);

*ρ*: Density of the permeate.

The decontamination factor (DF) for nuclides and nitric acid was calculated using the following expression:

(2) $$DF=\frac{C_{f}}{C_{p}}$$

where *C_f_* and *C_p_* represent the concentrations of nuclides or nitric acid in the feed and permeate solutions, respectively. Samples were collected and analyzed for nuclide concentrations and pH values in both the feed tank and condensate tank at the beginning and end of the experiment.

The rejection ratio (RF) of nuclides and nitric acid was calculated as follows:

(3) $$RF=\left(1-\frac{C_{p}}{C_{f}}\right)\times 100\%$$

with *C_f_* and *C_p_* being defined in the same manner as in Equation (2).

The calculation formula for relative flux (*J_r_*) is shown as follows:

(4) $$J_{r}=\frac{J}{J_{0}}$$

where *J* and *J*_0_ represent the membrane flux at a certain moment and the membrane flux using water, respectively.

### 2.3. Orthogonal Test Design

This report systematically investigates the operational parameters of VMD for radioactive wastewater treatment through orthogonal experimental design. Three critical process variables—feed solution temperature, vacuum pressure, and feed flow rate—were evaluated at four levels each using simulated radioactive solutions. Sr, Co, and Cs were used as the simulated nuclides and were dissolved in 0.1 mol·L^−1^ nitric acid; their concentration was 0.5 mg·L^−1^.

A total of 16 experimental runs were conducted according to the L_16_(4^5^) orthogonal array; the detailed factor-level assignments are presented in Table 1.

- Factor A (feed solution temperature): 40 °C, 50 °C, 60 °C, 70 °C.
- Factor B (vacuum pressure): 5 kPa, 15 kPa, 25 kPa, 30 kPa.
- Factor C (feed flow rate): 200 L·h^−1^, 300 L·h^−1^, 400 L·h^−1^, 500 L·h^−1^.

The orthogonal test matrix was designed to systematically evaluate interaction effects and to determine the optimal operating conditions for maximizing VMD system performance. A statistical analysis of the experimental results will be used to identify significant factors and their optimal combinations for radioactive wastewater treatment.

## 3. Results and Discussion

### 3.1. Selection of Operating Conditions for VMD

**Figure 3:**
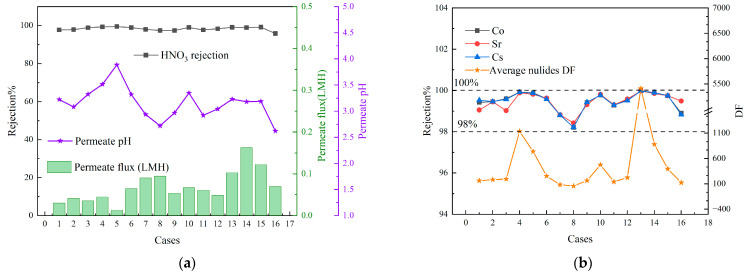
Results of orthogonal experiments. (**a**) Permeate flux and rejection rate of HNO_3_; (**b**) rejection rate and DF of nuclides.

The results of the orthogonal experiments, including the membrane flux, the nitric acid rejection rate, the rejection rates of the three nuclides (Sr, Co, and Cs), and the average DF of these nuclides, are shown in Figure 3. As depicted in the figure, the rejection rates of nitric acid and the nuclides remain consistently high across various operating conditions, with the nitric acid rejection rate reaching up to 99.4%. The pH value of the permeate is maintained within the range of 2.5–4. Additionally, the average DF of the nuclides exceeded 100 and can reach values greater than 1000. However, the overall membrane flux remains relatively low, with the highest recorded value being only 0.16 L·m^−2^·h^−1^.

Based on the orthogonal array design results, the optimal operating conditions were determined using the membrane flux, nitric acid rejection rate, and nuclide rejection rate as performance criteria. The detailed analysis procedures and results are presented in Table 2, Table 3 and Table 4. In these tables, the K-values represent the sum of experimental responses at each factor level, while T-values denote the normalized mean response (K-value divided by the number of replications).

The Range (R) is calculated as the difference between the maximum and minimum T-values for each factor. The statistical analysis indicated that the larger R-values correspond to a greater influence of the factor on system performance, while the higher T-values signify optimal operating levels.

Orthogonal test analysis revealed that the nuclide rejection rate remained consistently high across all experimental conditions, with no significant differences in influence being observed among the evaluated factors. Consequently, the nuclide rejection rate was excluded as a criterion for optimizing operating parameters. The feed solution temperature exerted the most substantial impact on membrane flux, while demonstrating a minimal effect on the nitric acid rejection rate. Therefore, temperature optimization prioritized flux enhancement, resulting in an optimal parameter of 70 °C. Conversely, the feed flow rate exhibited a significant influence on nitric acid rejection performance, leading to the selection of 500 L·h^−1^ as the optimal condition to maximize rejection efficiency. Vacuum pressure showed a moderate influence across all performance metrics, with consistent optimal levels being identified by all three evaluation criteria. The final optimized operating conditions were determined as A4B2C4, corresponding to the following:

- Feed temperature: 70 °C (A4);
- Vacuum pressure: 15 kPa (B2);
- Feed flow rate: 500 L·h^−1^ (C4).

### 3.2. Orthogonal Experiment Results Analysis

The results of the orthogonal test indicate that the VMD process is viable for treating acidic LLLW. Among the three operational parameters, temperature has the most significant impact on membrane flux. This can be attributed to the fundamental principle of MD, where volatile components in the feed solution are heated and transported across the membrane as vapor, which is driven by the vapor pressure difference between the two sides of the membrane [16]. Non-volatile substances, such as nuclides, do not volatilize into the gas phase and therefore cannot pass through the membrane pores, resulting in excellent rejection. Volatile substances such as water and nitric acid, on the other hand, have different partial pressures in the gas phase depending on temperatures and concentrations, allowing them to potentially permeate the membrane. The relative volatility of water is defined as the ratio of the molar fraction of water to that of nitric acid in the gas phase, divided by the ratio of the molar fraction of water to that of nitric acid in the liquid phase at equilibrium (α) [17].

**Figure 4:**
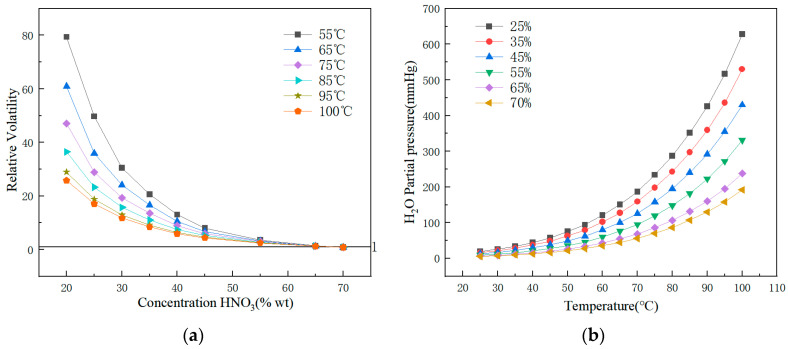
The relative volatility of water in nitric acid–water systems. (**a**) Relative volatility of water in the nitric acid–water system at different nitric acid concentrations. (**b**) Partial vapor pressure of water in the nitric acid–water system at different temperatures.

The partial pressure of nitric acid in the gas phase at different temperatures and mass fractions can be referenced from chemical engineering handbooks [18]. The relative volatility of water at different temperatures and nitric acid concentrations can be calculated to obtain a numerical value, as illustrated in Figure 4a. From Figure 4a, it is shown that when the nitric acid concentration approaches 70%, the relative volatility of water is ≤1. At this concentration, water and nitric acid volatilize to a similar extent and cannot be separated via MD. When the nitric acid concentration is lower, the relative volatility of water significantly exceeds that of nitric acid, and MD can be used to concentrate and separate the nitric acid. As shown in Figure 4b, in the nitric acid–water system, the partial pressure of water vapor increases exponentially with temperature [19]. This results in a larger vapor pressure difference across the membrane, enhancing the driving force and consequently increasing the membrane flux, which is also consistent with the results of the orthogonal test.

In addition to temperature, the vacuum degree on the permeate side is also an important factor influencing membrane flux. In an orthogonal experiment with four pressure gradients, the overall membrane flux was observed to be lower than the flux values reported in the literature. This difference may be attributed to the driving force on both sides being relatively low. In order to obtain a more optimal vacuum degree on the permeate side, further investigation was conducted on membrane flux under higher vacuum degrees. During the experiment, the feed solution temperature was maintained at 70 °C, while the flow rate was set to 500 L·h^−1^. Considering the influence of nitric acid in the solution, the vacuum degree on the permeation side was adjusted to 90 kPa with reference to the saturated vapor pressure of the 20% water–nitric acid solution at 70 °C, which is 26.7 kPa(a).

Three sets of experiments were conducted using the same device, and the test results are summarized in Table 5. The data indicate that increasing the vacuum pressure on the permeation side leads to a significant increase in flux, which stabilizes at 0.9 L·m^−2^·h^−1^. This phenomenon is mainly attributed to the fact that when the saturated vapor pressure of the hot-side feed liquid exceeds the pressure on the permeate side, the hot-side feed liquid is in a boiling state [20]. During boiling, the rate of vaporization is higher compared to when the feed solution is not boiling. Consequently, the membrane flux reaches its maximum value, demonstrating that, for optimal performance in VMD, the pressure on the permeate side should be lower than the saturated vapor pressure of water on the hot side.

### 3.3. Effect of Different Nuclide Feed Concentrations

This study also examines the effects of different nuclide feed concentrations on the flux and nuclide rejection rate of the VMD process. Experiments are proposed to be carried out using simulated feed solutions with three nuclide concentration gradients (0.5 mg·L^−1^, 5 mg·L^−1^, and 50 mg·L^−1^), and the simulated feed solution also contains approximately 0.07 mol·L^−1^ nitric acid.

**Figure 5:**
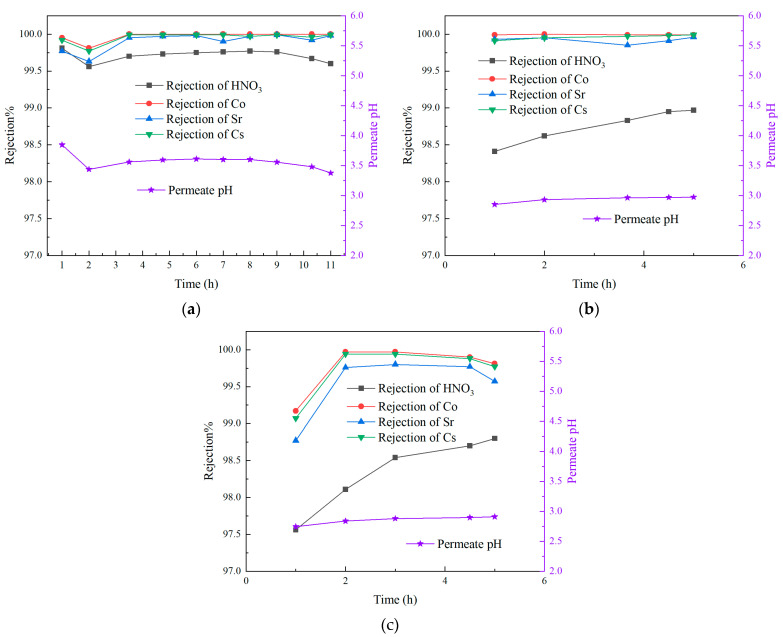
Variation in rejection rate with time at different nuclide feed concentrations. (**a**) Nitric acid 0.1 mol·L^−1^ and nuclide 0.5 mg·L^−1^; (**b**) nitric acid 0.1 mol·L^−1^ and nuclide 5 mg·L^−1^; (**c**) nitric acid 0.1 mol·L^−1^ and nuclide 0.5 mg·L^−1^.

Figure 5 illustrates the VMD retention rate of different nuclides in the feed solution at different nuclide concentrations, as well as the VMD retention effect on nitric acid. As shown in the figure, the VMD process demonstrates a good rejection effect on nuclides, with rejection rates exceeding 99.7% for all three nuclides; the DF ranges from 300 to 42,000. This corroborates that the rejection rate of non-volatile salts is independent of the concentration [21]. The rejection rate of nitric acid exhibits a decreasing trend as the nuclide concentration increases; however, the rejection rate remains above 98%, with the DF between 75 and 247. The increased salt concentration in the feed solution may deposit on the membrane surface or within pores, causing membrane wetting or mechanical damage [22]. As a volatile substance, nitric acid can more easily penetrate into the condensate once membrane fouling reduces membrane hydrophobicity, resulting in decreased rejection. Overall, these findings indicate that even at higher nuclide concentrations in the feed solution, the VMD process maintains a significant rejection rate for both nuclides and nitric acid.

During the experiment, as the feed solution was continuously concentrated, the nitric acid concentration also increased. As illustrated in Figure 4, following the increase in nitric acid concentration, the partial pressure of nitric acid in the hot-side steam correspondingly increased, potentially leading to an increase in the nitric acid content in the permeate. In this study, after five hours of operation, the nitric acid concentration in the feed solution reached approximately 0.1 mol·L^−1^. According to research by Tomaszewska [23], when the nitric acid concentration in the feed solution is below 0.8 mol·L^−1^ (55 °C), the nitric acid concentration in the permeate remains significantly less than 0.16 mol·L^−1^. When the nitric acid concentration in the feed solution approaches 3.1 mol·L^−1^, the nitric acid concentration in the permeate is approximately 0.8 mol·L^−1^. The results of this experiment indicate that, within the initial five-hour operational period, the nitric acid concentration in the permeate is below 0.001 mol·L^−1^. The results of Inga et al. [24] showed that when the feed solution was 1 mol·L^−1^ nitric acid at 60 °C, the nitric acid concentration in the permeate is 0.027 mol·L^−1^, indicating that membrane distillation can achieve a high concentration factor under low-temperature conditions.

**Figure 6:**
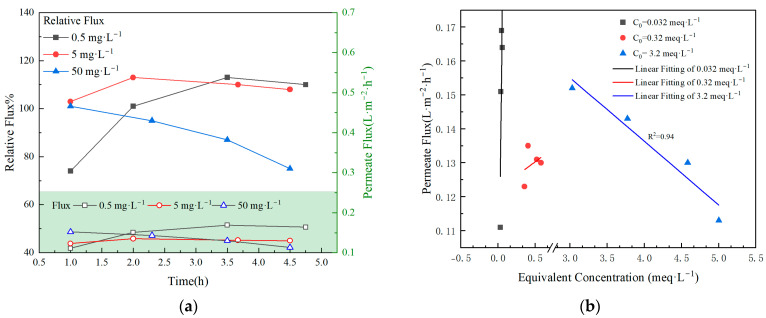
Variation in permeate flux at different nuclide feed concentrations. (**a**) Variation in permeate flux with time at different nuclide feed concentrations. (**b**) Variation in permeate flux at different nuclide feed concentrations.

Figure 6 illustrates the impact of varying nuclide concentrations in the feed solution on the membrane flux. Figure 6a presents the variation in membrane flux over time for different feed concentrations. During the five-hour operation period, the membrane flux remained generally stable. For the purpose of facilitating analysis, the three nuclides in the feed solution were converted to equivalent concentrations and summed. Specifically, when the individual nuclide concentrations were 0.5 mg·L^−1^, 5 mg·L^−1^, and 50 mg·L^−1^, the total equivalent concentration was 0.032 meq·L^−1^, 0.32 meq·L^−1^, and 3.2 meq·L^−1^, respectively. When the nuclide concentration in the feed is below 0.32 meq·L^−1^, there is no significant adverse effect on membrane flux. However, when the nuclide concentration exceeds 3.2 meq·L^−1^, a significant reduction in the membrane flux is observed. As the nuclide concentration increases, the membrane flux decreases by 25.6%, as shown in Figure 6b. A linear regression analysis of the experimental data reveals that when the equivalent nuclide concentration exceeds 3 meq·L^−1^, there is a negative correlation between nuclide concentration and membrane flux. Previous studies on membrane flux during the MD of high-salinity waste liquids typically involved much higher salt concentrations, often exceeding 1000 meq·L^−1^ [25]. At this time, the elevated salt content in the feed solution significantly reduced the vapor pressure of the solvent, consequently leading to a decline in membrane flux. Moreover, the high salt concentration aggravated the concentration polarization phenomenon [26].

However, for the acidic LLLW in the nuclear facilities, its specific activity is less than 4 × 10^6^ Bq·L^−1^ and it does not contain other inorganic salts. The typical specific activity of the nuclides in the solution is converted to an equivalent concentration of less than 7 × 10^−8^ meq·L^−1^. It is evident that the behavior of LLLW during the VMD process may differ from that of conventional high-salinity waste liquids. The experimental results indicate that at low nuclide concentrations, the membrane flux is essentially unaffected by nuclide concentration. VMD can achieve high concentration factors when treating LLLW.

### 3.4. Effect of TBP Concentration on Feed

This study further investigates the impact of TBP presence in the simulated feed solution on the flux and nuclide rejection rate of the VMD process. The simulated feed solution reports the following concentrations at 0.5 mg·L^−1^: nitric acid—0.1 mol·L^−1^; TBP—20 mg·L^−1^; and 100 mg·L^−1^.

**Figure 7:**
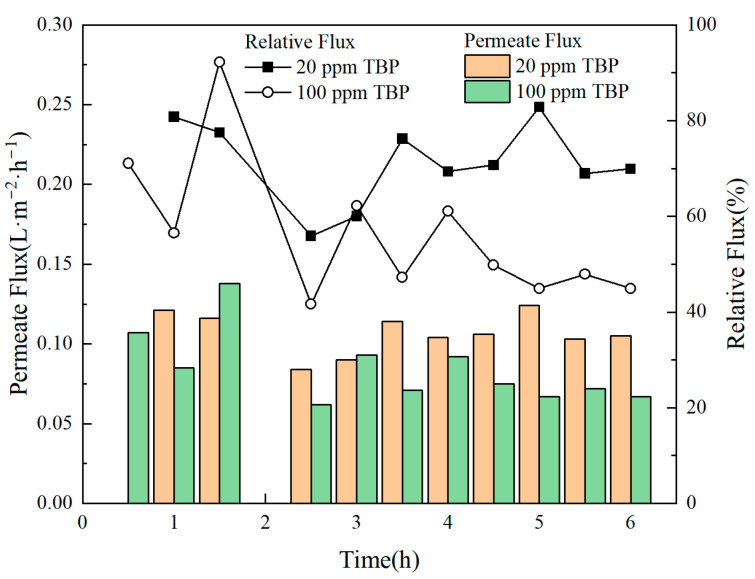
Variation in permeate flux at different TBP feed concentrations.

**Figure 8:**
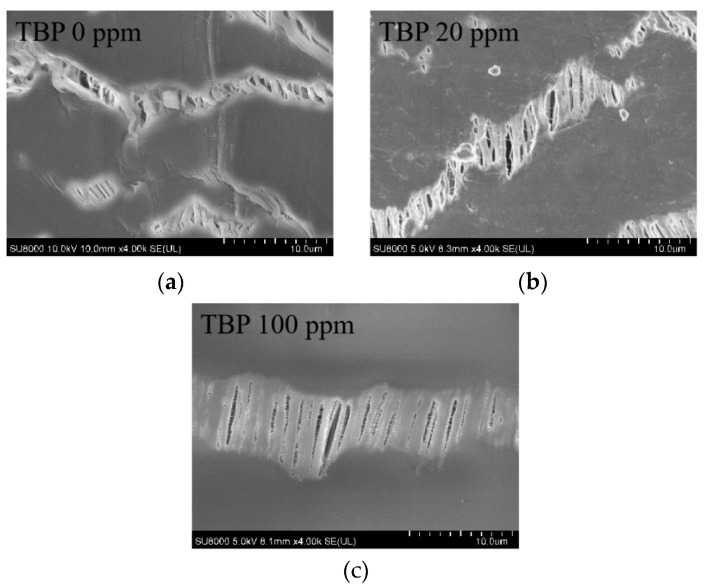
SEM characterization of the fouling membrane. (**a**) TBP 0.0 mg·L^−1^; (**b**) TBP 20 mg·L^−1^; (**c**) TBP 100 mg·L^−1^.

**Figure 9:**
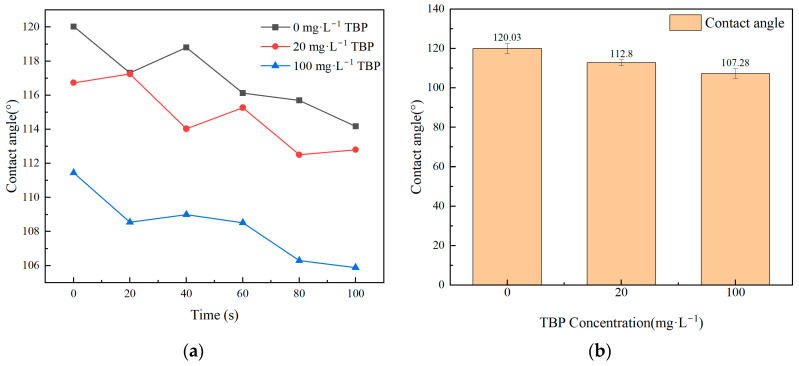
Effect of TBP on the hydrophobicity and wettability of membrane materials. (**a**) Variations in contact angle at different times; (**b**) variations in contact angle at different TBP concentrations.

The effect of different TBP concentrations on membrane flux is illustrated in Figure 7. As observed from the figure, in the presence of TBP, membrane flux fluctuated significantly over time, leading to membrane flux instability. The presence of TBP resulted in a decreasing trend in membrane flux. As the concentration of TBP increased, the degree of attenuation in membrane flux also increased, indicating the potential fouling of the membrane material by TBP. Table 6 illustrates the effects of varying TBP concentrations on the DF of nuclides and nitric acid. The results show that TBP reduces the membrane’s DF for both nitric acid and nuclides, although the rejection effect on nuclides remains relatively high. Figure 8 presents the SEM characterization of the membrane material contaminated by TBP, revealing no significant changes in surface morphology within a short period. Figure 9 displays the contact angle measurements of TBP-contaminated membranes. The static contact angle results indicate that TBP addition decreases the hydrophobicity of the membrane surface, leading to a reduced flux. TBP presence causes irreversible membrane surface hydrophilization, as shown by dynamic contact angle data, decreasing nuclide separation performance.

TBP is a widely utilized extractant that presents at trace levels in acidic LLLW. The factors contributing to membrane wetting by organic substances are multifaceted. Current research indicates that prevalent mechanisms include the following: (1) surfactant-like compounds, characterized by their inherently low surface tension, which reduce the surface tension of the solution, thereby decreasing the liquid entry pressure (LEP) and facilitating liquid penetration into membrane pores; (2) the adsorption and deposition of organic matter, which alter the hydrophobic properties of the membrane [27]. The TBP employed in this study is a water-insoluble extractant. However, as demonstrated by YU et al. [28], the surface tension of the aqueous phase decreases with increasing TBP concentration in a TBP–water system. The parameter governing membrane wetting propensity is the liquid entry pressure (LEP), which is determined by the liquid surface tension (γ), contact angle (θ) between the solution and membrane, geometric factor of membrane pores (B—a parameter related to pore structure), and maximum pore diameter (d_max_), as described by the following formula:

(5) $$LEP=-\frac{2B\gamma cos\theta }{d_{max}}$$

From this equation, in TBP-containing aqueous solutions, increasing the TBP concentration reduces the surface tension and contact angle, leading to a decrease in LEP. This facilitates liquid intrusion into membrane pores and diminishes membrane hydrophobicity. TBP may also adsorb onto the membrane surface, thereby modifying its hydrophobic properties. However, definitive conclusions regarding this mechanism necessitate future investigations into the chemical properties of the membrane surface.

Based on the results of membrane flux and DF analyses, combined with changes in the contact angle of the membrane material before and after operation, it is evident that the presence of TBP affects the hydrophilicity of the membrane, consequently impacting the efficiency of VMD. This effect intensifies as TBP concentration increases. Therefore, for LLLW containing TBP, it is advisable to implement a pretreatment process to remove TBP prior to using the VMD method.

## 4. Conclusions

The performance test of vacuum membrane distillation (VMD) has the following conclusions:

- Orthogonal experimental analysis revealed the optimal operational parameters for the VMD process as follows: a feed temperature of 70 °C, a permeate-side vacuum pressure of 90 kPa, and a feed flow rate of 500 L·h^−1^.
- The VMD process demonstrated a good performance in retaining nuclides and remains unaffected by variations in feed concentration. The rejection rate was consistently maintained at over 99.7%, with a DF exceeding 300. Additionally, the rejection rate of nitric acid remained above 98%. However, as the concentration of nuclides in the feed increased, the rejection rate exhibited a tendency to decrease.
- Under optimized operational parameters, the VMD process maintained a membrane flux of 0.9 L·m^−2^·h^−1^. At low nuclide concentrations in the feed solution, no significant negative effect on membrane flux was observed.
- The presence of TBP in the feed solution could lead to the contamination of the membrane material, thereby causing a reduction in membrane flux and a decline in DF. This issue was likely to become more obvious as the concentration of TBP increased.

VMD has emerged as a promising technology for the treatment of acidic low-level radioactive liquid waste, demonstrating a promising concentration and nuclide rejection performance. However, current commercial membranes still face challenges such as low flux and a tendency toward organic fouling. Future research directions should prioritize the development of advanced membrane materials to improve the comprehensive performance of VMD systems.

## Figures and Tables

**Table 1:** Orthogonal test parameters.

Cases	Testing Scheme	Factors		
		Feed Temperature (°C)	Vacuum Degree (kPa)	Flow Rate (L·h^−1^)
1	A1B1C1	40	5	200
2	A1B2C2	40	15	300
3	A1B3C3	40	25	400
4	A1B4C4	40	30	500
5	A2B1C2	50	5	300
6	A2B2C1	50	15	200
7	A2B3C4	50	25	500
8	A2B4C3	50	30	400
9	A3B1C3	60	5	400
10	A3B2C4	60	15	500
11	A3B3C1	60	25	200
12	A3B4C2	60	30	300
13	A4B1C4	70	5	500
14	A4B2C3	70	15	400
15	A4B3C2	70	25	300
16	A4B4C1	70	30	200

**Table 2:** Optimum operation parameters determined by membrane flux.

Parameters	Factors	K	T	Range	Rank	Optimum Operation Parameters
Feed temperature (°C)	A1	0.1505	0.0376	0.0766	Feed temperature > Vacuum degree > Flow rate	A4B2C3
	A2	0.2611	0.0653			
	A3	0.2298	0.0574			
	A4	0.4570	0.1143			
Vacuum degree (kPa)	B1	0.1985	0.0496	0.0342		
	B2	0.3353	0.0838			
	B3	0.3076	0.0769			
	B4	0.2569	0.0642			
Flow rate (L·h^−1^)	C1	0.2247	0.0562	0.0303		
	C2	0.2243	0.0561			
	C3	0.3457	0.0864			
	C4	0.3037	0.0759			

**Table 3:** Optimum operation parameters determined by HNO_3_ rejection rate.

Parameters	Factors	K	T	Range	Rank	Optimum Operation Parameters
Feed temperature (°C)	A1	3.9398	0.9849	0.0035	Flow rate > Vacuum degree > Feed temperature	A2B2C4
	A2	3.9403	0.9851			
	A3	3.9263	0.9816			
	A4	3.9319	0.9830			
Vacuum degree (kPa)	B1	3.9392	0.9848	0.0096		
	B2	3.9490	0.9873			
	B3	3.9396	0.9849			
	B4	3.9104	0.9776			
Flow rate (L·h^−1^)	C1	3.9035	0.9759	0.0132		
	C2	3.9503	0.9876			
	C3	3.9283	0.9821			
	C4	3.9561	0.9890			

**Table 4:** Optimum operation parameters determined by nuclide rejection rate.

Parameters	Factors	K	T	Range	Rank	Optimum Operation Parameters
Feed temperature (°C)	A1	398.1039	99.5260	0.5302	Flow rate > Vacuum degree > Feed temperature	A2B2C4
	A2	396.5675	99.1419			
	A3	398.0212	99.5053			
	A4	398.6885	99.6721			
Vacuum degree (kPa)	B1	398.5627	99.6407	0.4776		
	B2	398.7356	99.6839			
	B3	397.2574	99.3143			
	B4	396.8253	99.2063			
Flow rate (L·h^−1^)	C1	397.3109	99.3277	0.4129		
	C2	398.6146	99.6537			
	C3	396.9630	99.2407			
	C4	398.4925	99.6231			

**Table 5:** Results of flux and HNO_3_ rejection (%) in high vacuum degree conditions.

Cases	Flux (L·m^−2^·h^−1^)	HNO_3_ Rejection (%)	Permeate pH
1	0.953	99.62	3.44
2	0.913	99.71	3.56
3	0.930	98.91	3.16
Average	0.932 ± 0.0163	99.41 ± 0.358	3.39 ± 0.168

**Table 6:** Variation in nitric acid and nuclide rejection rate at different TBP feed concentrations.

Feed Composition	HNO_3_ DF	Co DF	Sr DF	Cs DF
0 mg·L^−1^ TBP	247	41,733	5853	10,317
20 mg·L^−1^ TBP	135	1332	3010	822
100 mg·L^−1^ TBP	66	482	572	383

## Data Availability

The original contributions presented in this study are included in the article. Further inquiries can be directed to the corresponding author.
